# Tuning the structural and electronic properties and chemical activities of stanene monolayers by embedding 4d Pd: a DFT study

**DOI:** 10.1039/c9ra01472a

**Published:** 2019-05-22

**Authors:** Amirali Abbasi

**Affiliations:** Molecular Simulation Laboratory (MSL), Azarbaijan Shahid Madani University Tabriz Iran a_abbasi@azaruniv.ac.ir; Computational Nanomaterials Research Group (CNRG), Azarbaijan Shahid Madani University Tabriz Iran; Department of Chemistry, Faculty of Basic Sciences, Azarbaijan Shahid Madani University Tabriz Iran

## Abstract

We have thoroughly investigated the interaction of some gas molecules (CO, NO, N_2_O and NH_3_) with Pd-decorated stanene nanosheets using density functional theory calculations. In this regard, we have considered three patterns for embedding Pd into the stanene monolayer, and then placed gas molecules on the Pd-decorated systems. Initially, we have optimized the structure of the Pd-decorated stanene to obtain its electronic properties. The charge density difference plot of the Pd-decorated system represents the accumulation of charge density on the adsorbed Pd atom. The adsorption energies, density of states, charge density differences and electronic band structures were analyzed in detail to fully exploit the gas sensing performance of Pd-decorated stanene systems. All the studied gas molecules form covalent bonds with the embedded Pd atom, which indicates the strong interaction between gas molecules and Pd-decorated stanene. The adsorption of gas molecules on pattern-III Pd-embedded stanene monolayers is more energetically favorable than that on the pattern-I and pattern-II ones. Besides, band structure calculations indicate changes in the electronic structure of the studied systems upon gas adsorption. Based on Mulliken charge analysis, the positive charge transfer occurred from the gas molecules to the Pd-decorated stanene systems. The results of this paper could provide a useful basis for materials scientists to design and modify novel sensing materials based on Pd-decorated stanene monolayers.

## Abstract 2

The structural, electronic and chemical activities of stanene monolayers can be effectively tuned by introducing Pd atoms.

## Graphical abstract

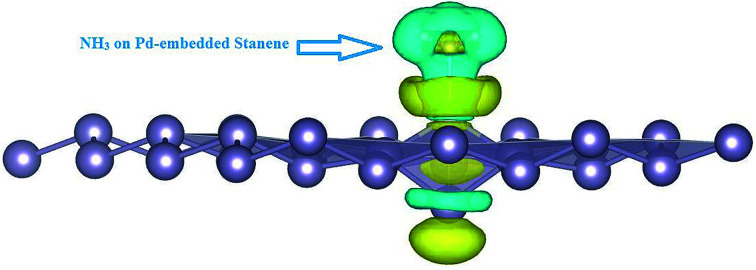

## 1. Introduction

In the past few decades, graphene as one of the most popular members of the two-dimensional (2D) materials family has attracted tremendous interest because of its particular mechanical and electronic properties.^1^ Graphene has been utilized in an extensive range of applications such as integrated circuits, transparent conducting electrodes, and hydrogen storage materials.^2–6^ Recently, researchers have demonstrated the superior gas sensing capability of graphene, which could be mostly attributed to its high sensitivity and selectivity towards gas molecules.^7–10^ Experimentally, it has been reported that the graphene shows high sensitivity to some gas molecules such as NH_3_, CO and NO_2_.^6^ Graphene has a planar structure, and represents a very weak interaction with gas molecules as confirmed by some experimental^11^ and theoretical^12,13^ works. Thus, the adsorbates were only physisorbed on the surface of pure graphene, and this weak interaction is mainly characterized as van der Waals interaction.

The weak physisorption of gas molecules on the pure graphene significantly limits its sensing capability. To tackle this issue, some efficient strategies have been developed to increase the sensitivity of graphene to gas molecules such as the introduction of point defects or substitutional elemental doping into graphene.^14–17^ For instance, performing theoretical calculations, Dai *et al.* proposed that B- and S-doped graphene can react with NO and NO_2_ molecules more strongly.^14^ Nevertheless, the doping method cannot act as an effective method to enhance the sensitivity of pure graphene as only a very small portion of graphene atoms was substituted by reactive dopants. In order to overcome this problem, scientists have tried to search for other graphene-like materials with a high surface to volume ratio, which show high reactivity with gas molecules. As one of these 2D graphene-like materials, silicene, a layered hexagonal structure of silicon atoms,^18^ has been theoretically predicted^19–22^ and fabricated in the laboratory for practical applications.^23–30^ Stanene is also another 2D counterpart of graphene, and has attracted significant attention during the past few years. Stanene is a monolayer of hexagonally arranged tin atoms, which has been synthesized through molecular beam epitaxial method.^31^

Density functional theory calculations demonstrated that stanene is a gapless material and it shows a Dirac cone around the Fermi level. A sizeable band gap of 0.1 eV was achieved when the effect of spin–orbit coupling (SOC) was taken into account.^32–34^ Thus, the electronic properties of stanene can be modulated through different methods. Tuning the electronic and magnetic properties of 2D materials is very important procedure, which sheds light on the extensive applications of 2D materials in nanoelectronic devices. Recently, some theoretical works have been conducted on the adsorption of atomic/molecular agents on the surface of 2D materials, which greatly modifies the electronic structures.^35,36^ For example, Valencia *et al.* studied the nature of magnetism caused by the adsorption of transition metals on graphene.^37^ Toxic gas sensing has an eminent significance with great impacts on public health and environmental protection. Following this, 2D material based gas sensors are highly desired due to their great surface to volume ratio. Sensors constructed from 2D graphene,^38,39^ MoS_2_,^40^ and phosphorene^41,42^ have aroused tremendous attentions and the reason for this is because they possess low content of noise and low power consumption. There are a quite variety of 2D material based sensors including graphene, silicene, germanene *etc.* Among them, stanene has been considered to be a good candidate for gas sensing purpose due to its buckled structure, which greatly increases its reactivity compared to the other graphene-like materials. Recently, Chen *et al.* investigated the adsorption of small gas molecules on stanene using DFT calculations.^43^

In our previous works, we have also examined the adsorption behaviors of two-dimensional stanene monolayers and TiO_2_ based nanoparticles, and hybrid nanocomposites composed of stanene and TiO_2_.^44–53^ Zhou *et al.* studied the adsorption of gas molecules on transition metal embedded graphene using first principles calculations.^54^ The sensitivity and selectivity of some nanostructures such as graphene/gold nanointerfaces, C_3_N, and hexagonal YN have been also addressed in some works.^55–57^

In this work, we performed a DFT study to investigate the effects of the adsorption of gas molecules on the electronic properties of Pd-decorated stanene monolayers. The charge density differences, electronic band structures and projected density of states were analyzed in detail. Besides, the charge transfer between gas molecules and Pd-decorated stanene systems was evaluated based on the Mulliken charge analysis method.

## 2. Computational methods and models

All the calculations were performed based on the density functional theory (DFT),^58,59^ with the help of SIESTA code.^60^ To calculate the exchange–correlation energy, the generalized gradient approximation (GGA) in the formalism of Perdew–Burke–Ernzerhof (PBE) was applied.^61^ To perform density functional theory calculations, plane wave Pseudo-potential method was considered. To optimize the structures and electronic structure calculations, the Mesh cutoff was set to 200 Ry. GDIS program was employed to help in the construction of supercell models of the considered nanosheets.^62^ We chose the norm-conserving Troullier–Martins pseudopotential^63^ and double-zeta basis sets plus polarization functions (DZP). The selected unit cells were sampled using the Monkhorst–Pack *k*-point sampling pattern of 10 × 10 × 1 *k*-points,^64^ which led to the converged results. For band structure calculations in the first Brillouin zone, the *k*-point path is considered to be Ã–K–M–K–Ã. Geometry relaxations were followed using the conjugated-gradient method until the residual forces acting on each atoms were less than 0.02 eV Å^−1^. We have also employed the VESTA (visualization for electronic and structural analysis) program for visualization of the charge density diagrams and all geometric structures.^65^

For the calculations concerning stanene monolayer, we established a 4 × 4 supercell of stanene, and then investigated the adsorption of Pd atoms on the surface. At the first step, we studied the adsorption of Pd atom on three different sites of stanene, namely the hollow (H), valley (V) and top (T) sites, and calculated the electronic band structures. Then, we have placed the gas molecule on the three patterned Pd-embedded stanene systems. The adsorption energy (Δ*E*_ad_) of the Pd atom on the stanene surface is calculated as the following equation:

1 Δ*E*_ad_ = *E*_Pd-stanene_ − *E*_Pd_ − *E*_stanene_

where *E*_Pd-stanene_ is the total energy of the stanene monolayer with adsorbed Pd atom, while *E*_Pd_ and *E*_stanene_ represent the total energies of the isolated Pd atom and pristine stanene monolayer, respectively. Furthermore, we examined the adsorption of CO, NO, N_2_O and NH_3_ gas molecules on the Pd-decorated stanene monolayers. Similarly, the adsorption energy of these gas molecules on the Pd-decorated stanene was evaluated based on the following equation:

2 Δ*E*_ad_ = *E*_complex_ − *E*_gas molecule_ − *E*_Pd-stanene_

again, *E*_complex_, *E*_Pd-stanene_, and *E*_gas molecule_ represent the total energies of the Pd-decorated stanene monolayer with and without the adsorbed gas molecules, and free gas molecule, respectively.

## 3. Results and discussion

### 3.1. Optimized structure and electronic properties of Pd-embedded stanene

**Fig. 1:**
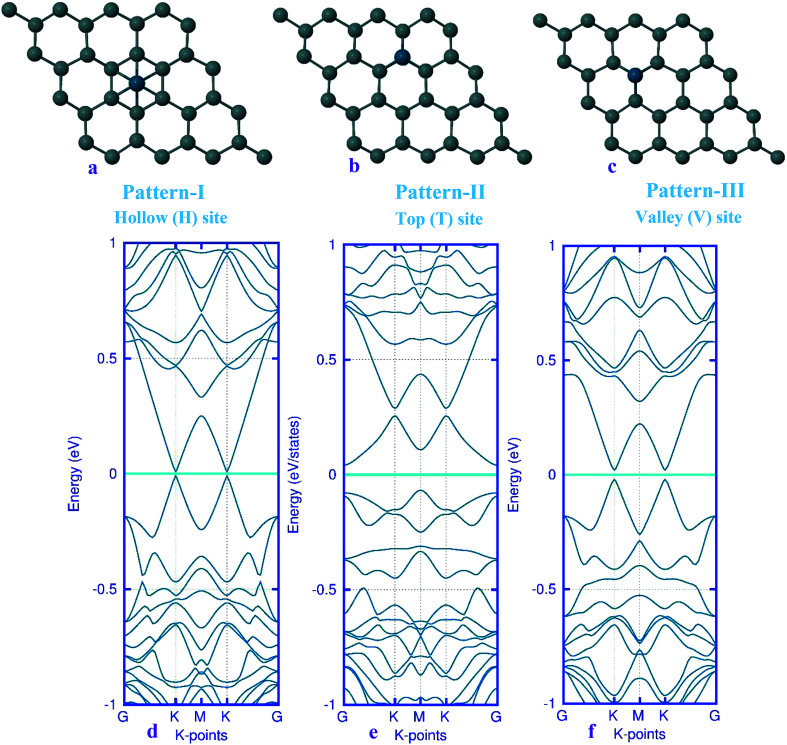
Optimized structure of the three patterns of Pd-decorated stanene monolayers along with the electronic band structure plots.

**Fig. 2:**
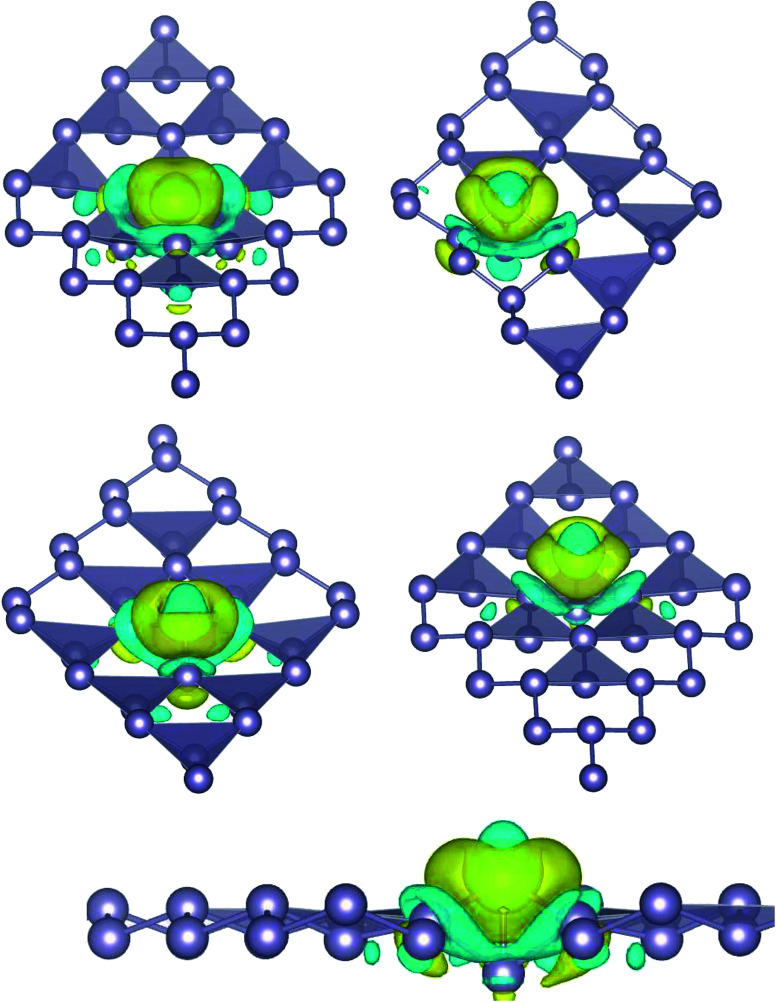
Isosurface plot of the electron charge density difference for three patterns of Pd-decorated stanene monolayers with the isovalue of ±0.0002 a.u.

Here, we studied the effects of the embedding of Pd atom on the structural and electronic properties of stanene monolayers. To this end, three patterns for Pd adsorption on the stanene surface were considered, namely hollow (H) site, top (T) site and valley (V) site, as shown in Fig. 1a–c. To explore the electronic properties of the Pd-embedded system, we have presented the band structure plots of the Pd-decorated stanene monolayers (Fig. 1d–f). As can be seen, all three cases represent semiconductor behavior, which indicates their suitability for gas sensing applications. The adsorption energies were calculated to gain more insights into the stability of the obtained Pd-embedded structures. For the adsorption of Pd atom on the stanene system, the most stable configuration is that Pd atom was adsorbed on the hollow (H) site above the center of a hexagon and coordinated to six Sn atoms. The adsorption energy of Pd on the H site (−6.80 eV) is higher (more negative) than that on the T (−6.09 eV) and V (−6.31 eV) sites, implying that the Pd adsorption on the H site is more energetically favorable than the adsorption on the other sites. In order to further examine the electronic interaction between the Pd atom and the stanene sheet, we calculated the charge density difference of Pd embedded on the stanene monolayer (Fig. 2). The charge density difference was defined as the following formula:

3 Δ*ρ* = *ρ*_(stanene+Pd)_ − *ρ*_(stanene)_ − *ρ*_(Pd)_

where, *ρ*_(stanene+Pd)_, and *ρ*_(stanene)_ denote the total charge densities of the stanene sheet with and without embedded Pd, respectively and *ρ*_(Pd)_ is the charge density of an isolated Pd atom. It can be seen from Fig. 2 that the charges were largely collected over the adsorbed Pd atom. Additionally, the concentration of electronic density between the Pd and Sn atoms indicates that the embedding of Pd on the stanene sheet is a typical chemisorption.

### 3.2. Adsorption of gas molecules on pattern-I Pd-decorated stanene monolayer

**Fig. 3:**
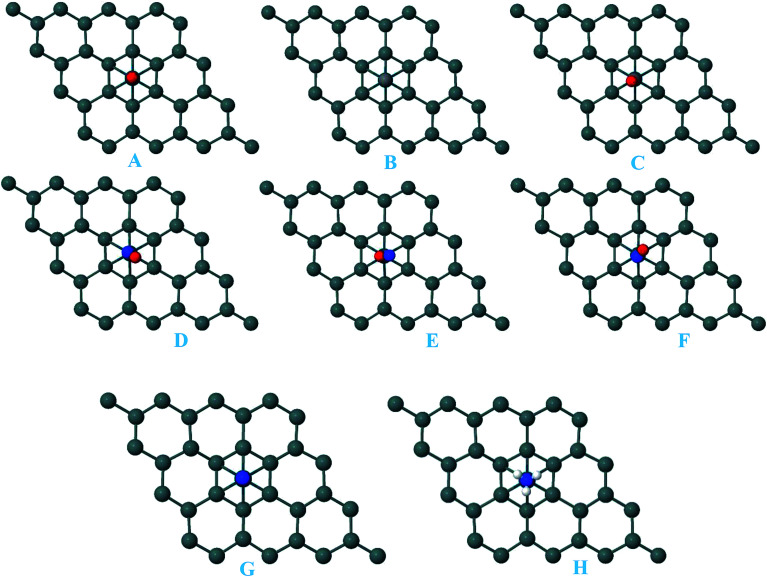
Optimized geometry configurations of the pattern-I Pd-decorated stanene monolayers with adsorbed CO, NO, N_2_O and NH_3_ molecules.

**Fig. 4:**
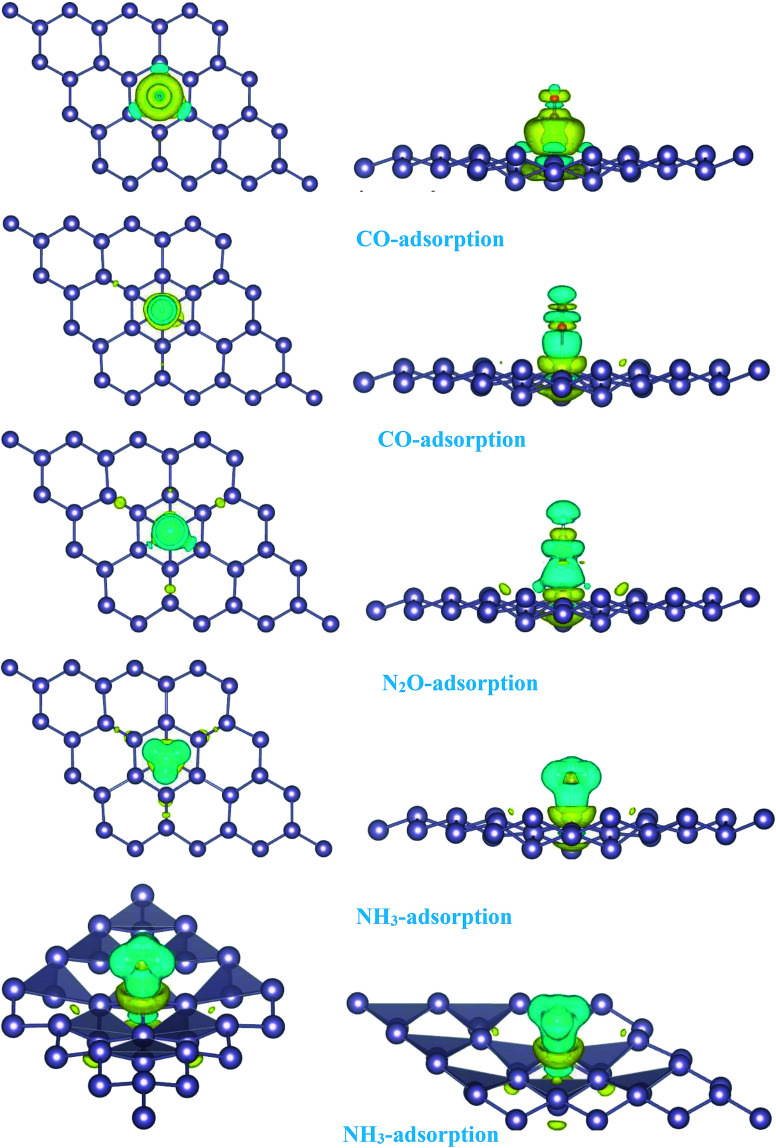
Isosurface plots of the electron charge density difference for pattern-I Pd-decorated stanene monolayers with adsorbed CO, N_2_O and NH_3_ molecules (isovalue is ±0.0002 a.u).

**Fig. 5:**
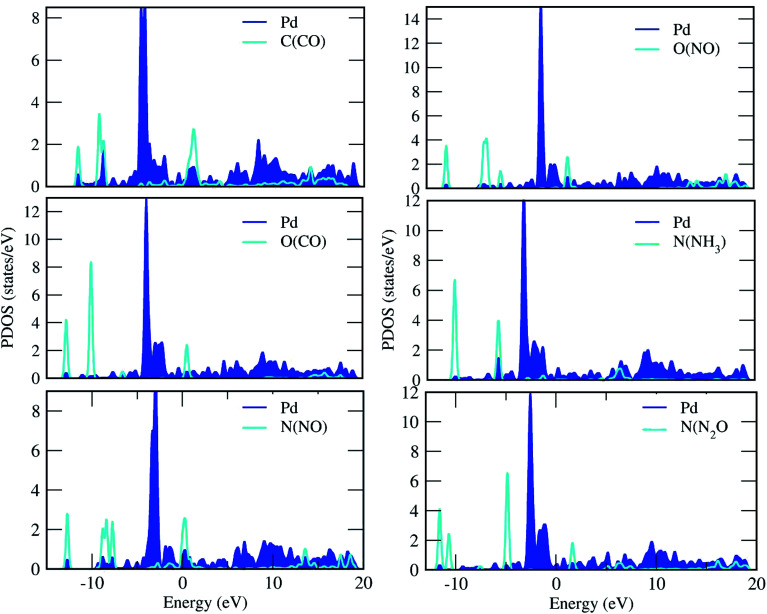
Projected density of states of the C, O, N and Pd atoms for the adsorption of gas molecules on the Pd-decorated stanene monolayers.

For the adsorption of gas molecules on Pd-decorated stanene monolayers, we considered various adsorption configurations to search for the most stable adsorption positions of gas molecules above the stanene surface. As mentioned above, we have modeled three patterns for Pd embedding into the stanene system, and then studied the adsorption of gas molecules on these Pd-decorated monolayers. This section presents the interaction of gas molecules with pattern-I Pd-decorated stanene monolayer, in which the Pd atom was embedded on the hollow (H) site of stanene hexagon. Total energies were calculated for various adsorption sites and molecule orientations to evaluate the adsorption energies of the configurations. Fig. 3 shows the optimized geometry configurations of CO, NO, N_2_O, and NH_3_ molecules on Pd-decorated stanene monolayers. As can be seen from this figure, configurations A–C represent the adsorption of CO molecule on the Pd-decorated stanene, and configurations D-F show the interaction of NO molecule with Pd-decorated monolayers. Configurations G and H also present the adsorption configurations for N_2_O adsorbed and NH_3_ adsorbed Pd-embedded stanene systems, respectively. For CO adsorption on the surface, configuration A shows the adsorption of CO by its carbon atom, whereas configuration B represents CO adsorption by its oxygen atom on the surface. In the case of NO adsorption, configurations D and E represent the interaction of NO molecule by its nitrogen and oxygen atoms with the Pd-decorated stanene, respectively. As can be seen from this figure, both CO and NO molecules chemisorb on the Pd-embedded stanene monolayer since their constituent atoms strongly bind to the Pd atom. For N_2_O adsorption (configuration G), and NH_3_ adsorption (configuration H), we have only presented one configuration as the most stable site. Table 1 summarizes the bond lengths and adsorption energies for various gas molecules adsorption on the Pd-decorated stanene monolayers. The smaller distance between the interacting atoms indicate the strong interaction between gas molecules and Pd-decorated stanene systems. For CO adsorption on the surface, the adsorption energies of configurations A and B were calculated to be −1.76 eV and −0.5 eV, respectively. This indicates that the adsorption of CO molecule through its carbon atom on the surface is more energetically favorable than that through its oxygen atom. Therefore, CO molecule strongly interacts with Pd-decorated stanene through its carbon atom. In the case of NO adsorption, the adsorption energies for configurations D and E were calculated to be −2.15 eV and −1.05 eV, respectively, indicating that the binding of nitrogen atom to the surface is more favorable in energy than that of oxygen atom. Among the four studied gas molecules, N_2_O presents the weakest adsorption on the surface with the calculated adsorption energy of about −0.44 eV and distance of 2.42 Å between the oxygen and Pd atoms. Thus, the higher adsorption energy gives rise to the smaller distance between the gas molecules and Pd-decorated stanene monolayers. To further investigate the electronic properties of the Pd-decorated stanene monolayers upon the adsorption of gas molecules, we have calculated their charge density differences, as shown in Fig. 4. It can be seen from this figure that charges were accumulated on the adsorbed gas molecules. Besides, the accumulation of electron charge density at the middle of the Pd atom and substrate's atoms represent the formation of chemical bonds between them. This formation of covalent bonds was also confirmed by the projected density of states of the interacting atoms (Fig. 5). It can be seen that there is large electron orbital overlap between CO, NO and NH_3_ gas molecules and Pd-decorated stanene, confirming this fact that strong chemisorption occurs in these cases.

**Table 1:** Adsorption energies (in eV), Mulliken charge transfers (in e) and distances (in Å) between the nearest interacting atoms for different gas molecules adsorbed on the patterned Pd-decorated stanene monolayers

Configuration	Adsorption energy	Distance (*d*)	Charge transfer (Δ*Q*)
**Pattern-I**			
A	−1.76	(Pd–C) 1.98	0.23
B	−0.50	(Pd–O) 2.34	0.12
C	−1.77	(Pd–C) 1.98	0.23
D	−2.15	(Pd–N) 1.97	0.18
E	−1.05	(Pd–O) 2.16	0.14
F	−2.14	(Pd–N) 1.97	0.18
G	−0.44	(Pd–O) 2.42	0.11
H	−1.03	(Pd–N) 2.32	0.23

**Pattern-II**			
A	−2.02	(Pd–C) 1.96	0.23
B	−0.66	(Pd–O) 2.23	0.13
C	−2.02	(Pd–C) 1.97	0.23
D	−2.48	(Pd–N) 1.92	0.16
E	−1.27	(Pd–O) 2.08	0.11
F	−2.49	(Pd–N) 1.93	0.16
G	−0.60	(Pd–O) 2.27	0.13
H	−1.40	(Pd–N) 2.26	0.26

**Pattern-III**			
A	−2.19	(Pd–C) 1.95	0.22
B	−0.73	(Pd–O) 2.22	0.13
C	−2.18	(Pd–C) 1.96	0.22
D	−2.50	(Pd–N) 1.90	0.16
E	−1.30	(Pd–O) 2.06	0.10
F	−2.52	(Pd–N) 1.91	0.16
G	−0.64	(Pd–O) 2.26	0.13
H	−1.40	(Pd–N) 2.25	0.26

**Fig. 6:**
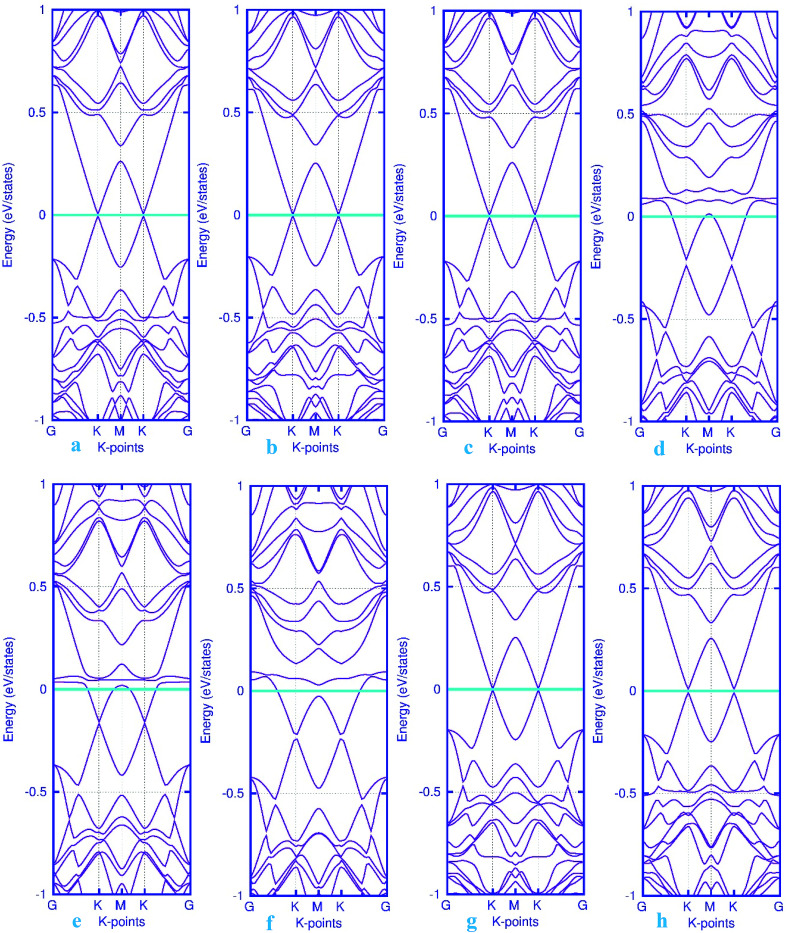
Electronic band structure plots for pattern-I Pd-decorated stanene monolayers with adsorbed CO (a–c) NO (d–f), N_2_O (g) and NH_3_ (h) molecules. The Fermi level is shifted to zero and indicated by a cyan solid line.

The band structure calculations were also conducted in this work to fully analyze the changes in the electronic properties caused by the adsorption of gas molecules. As mentioned above, pattern-I Pd-embedded stanene shows a semiconductor characteristics with a calculated band gap value of about 18 meV. The band structure plots for CO, NO, N_2_O and NH_3_ adsorbed Pd-decorated stanene monolayers were displayed in Fig. 6. As can be seen form Fig. 6a–c, the electronic band structure of the Pd-decorated stanene system is basically unaltered after CO adsorption, and the system remains still semiconductor with a Dirac cone located at K point. In case of NO adsorption (Fig. 6d–f), the NO adsorbed system exhibits metallic behavior compared to the semiconductor property in the bare Pd-decorated system. It can be seen from this figure that the Fermi level shifts to the conduction band edge after NO adsorption. In case of N_2_O and NH_3_ adsorption, the relevant band structure plots were also depicted in Fig. 6g and h. This figure shows that both N_2_O and NH_3_ adsorbed Pd-decorated stanene monolayers exhibit semiconductor property.

### 3.3. Adsorption of gas molecules on pattern-II Pd-decorated stanene monolayer

**Fig. 7:**
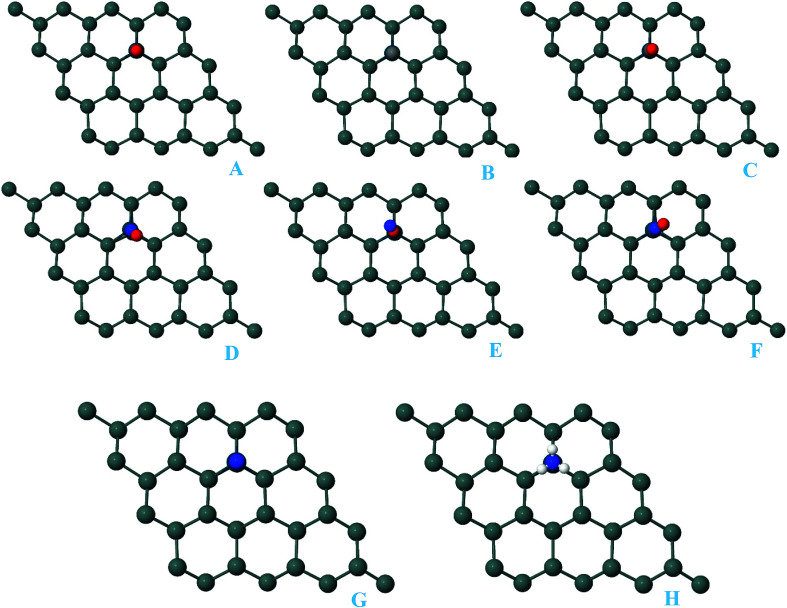
Optimized geometry configurations of the pattern-II Pd-decorated stanene monolayers with adsorbed CO, NO, N_2_O and NH_3_ molecules.

In this section, we investigated the adsorption of CO, NO, N_2_O and NH_3_ molecules on pattern-II Pd-decorated stanene monolayers. As described above, in this patter, Pd was placed above the top Sn atom of stanene monolayer and the whole system was relaxed. After relaxation, Sn atom extrudes outwards form the plane and the Pd atom occupies its place. Fig. 7 presents the most stable adsorption configurations of gas molecules on pattern-II Pd-decorated stanene monolayers. As can be seen, all gas molecules strongly bind to the Pd atom on the surface, indicating the chemisorption process. For CO adsorption, we have considered three configurations, as shown by complexes A–C in Fig. 7. Configurations A and B represent the adsorption of CO molecule by its carbon and oxygen atoms, respectively, and configuration C shows a bridge site for CO orientation above the surface, which finally results in a complex with carbon atom connected to the Pd atom after relaxation. This indicates the carbon atom of CO molecule tends to be preferentially adsorbed on the Pd-decorated stanene. The adsorption energies for configurations A–C were calculated to be −2.02 eV, −0.66 eV and −2.02 eV, respectively. The results suggest that the adsorption of CO on the surface by its carbon atoms is more energetically favorable than that by its oxygen atom. In case of NO adsorption, configurations D–F show the adsorption of NO by its nitrogen atom, the adsorption by oxygen atom and bridge adsorption, respectively. The calculated adsorption energies and bond lengths were listed in Table 1. The higher adsorption energy of configuration D (−2.48 eV) comparing with configuration E (−1.27 eV) indicates that the adsorption of NO by its nitrogen atom on the surface is more favorable in energy than that by its oxygen atom. In case of N_2_O and NH_3_ adsorption, the calculated adsorption energies are −0.60 eV and −1.40 eV, respectively. As can be seen, N_2_O molecule weakly interacts with Pd-decorated stanene monolayer compared to the other gas molecules, which is manifested by its relatively higher distance towards the Pd atom (2.27 Å).

**Fig. 8:**
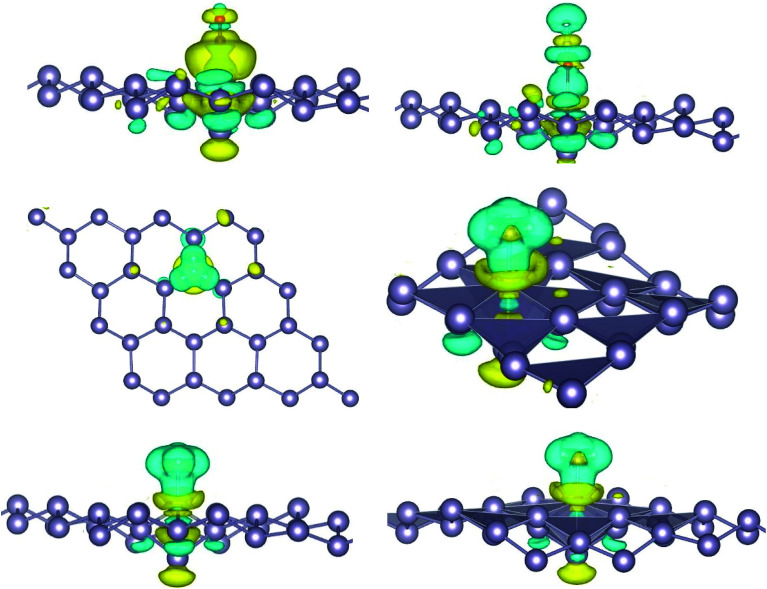
Isosurface plots of the electron charge density difference for pattern-II Pd-decorated stanene monolayers with adsorbed CO, N_2_O and NH_3_ molecules (isovalue is ± 0.0002 a.u).

**Fig. 9:**
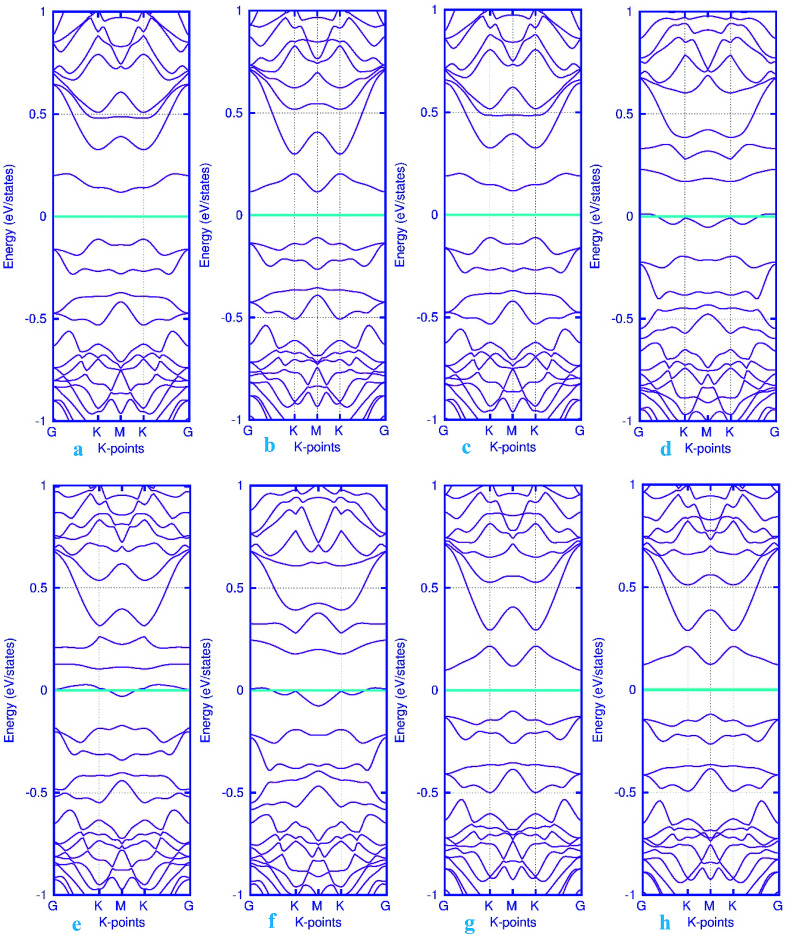
Electronic band structure plots for pattern-II Pd-decorated stanene monolayers with adsorbed CO (a–c) NO (d–f), N_2_O (g) and NH_3_ (h) molecules. The Fermi level is shifted to zero and indicated by a cyan solid line.

To investigate the effects of gas molecules on the electronic properties of Pd-decorated stanene monolayers, the charge density difference plots for CO, N_2_O and NH_3_ adsorbed systems were calculated and depicted in Fig. 8. As can be seen from this figure, the charges were mainly accumulated on the gas molecules after the adsorption process. To gain more insight into the chemisorption of gas molecules, their electronic band structures were calculated, as depicted in Fig. 9. Pattern-II Pd-doped stanene shows a semiconductor behavior with an indirect band gap. After the adsorption, the electronic properties of the system were substantially changed. Interestingly, all gas adsorbed Pd-decorated stanene systems represent semiconductor characteristics with a Fermi level located between the VBM and CBM. The sizeable band gap for CO adsorbed systems (Fig. 9a–c) exhibits its superior semiconductor property compared to the other systems.

### 3.4. Adsorption of gas molecules on pattern-III Pd-decorated stanene monolayer

**Fig. 10:**
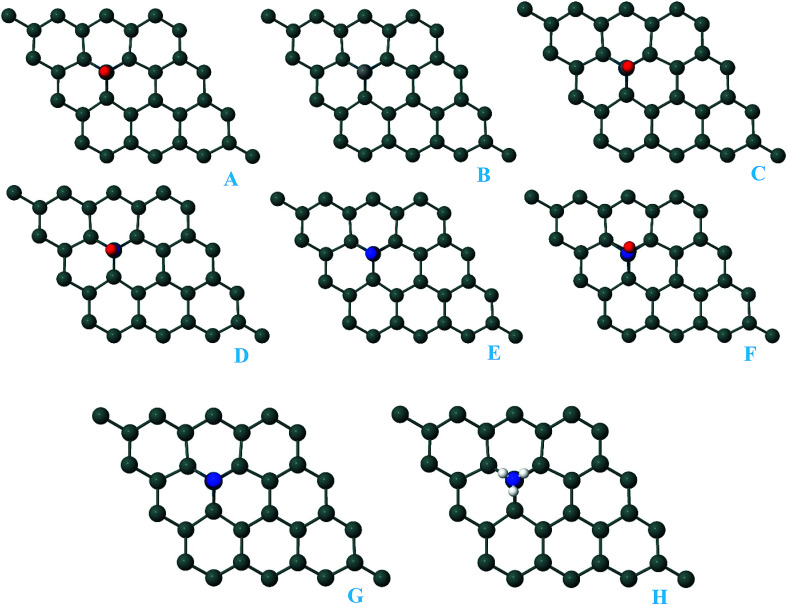
Optimized geometry configurations of the pattern-III Pd-decorated stanene monolayers with adsorbed CO, NO, N_2_O and NH_3_ molecules.

In order to further explore the adsorption capacity of the Pd-decorated stanene monolayers for gas molecules, we also investigated the adsorption of CO, NO, N_2_O and NH_3_ gases on the pattern-III Pd-decorated stanene systems (valley site adsorption). The optimized structures of the gas adsorbed Pd-decorated stanene monolayers were shown in Fig. 10.

**Fig. 11:**
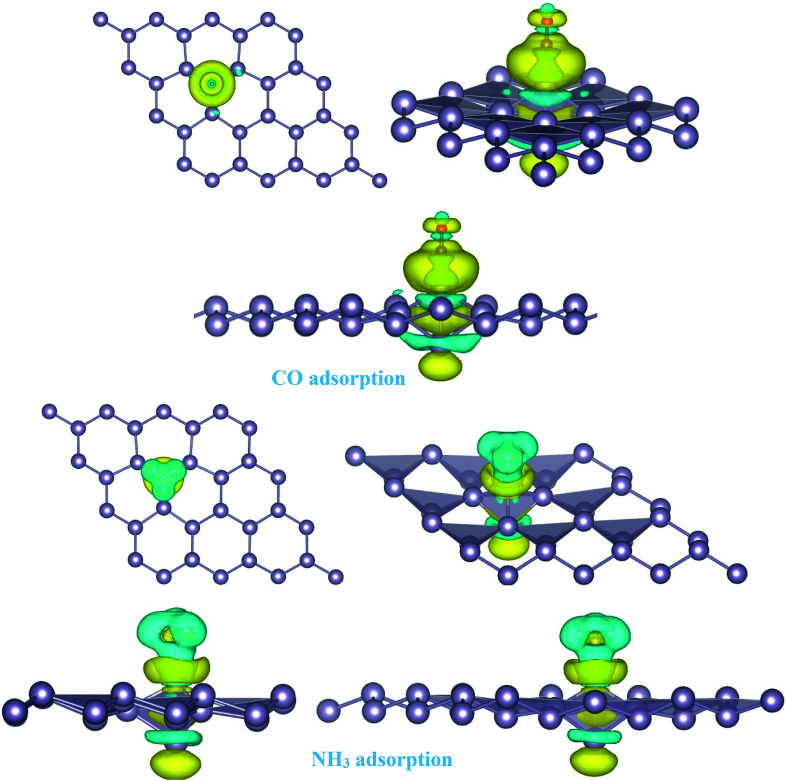
Isosurface plots of the electron charge density difference for pattern-III Pd-decorated stanene monolayers with adsorbed CO and NH_3_ molecules (isovalue is ± 0.0002 a.u).

The adsorption energy results were listed in Table 1. Similarly, the adsorption of CO molecule on the surface by its carbon atom (−2.19 eV) is more energetically favorable than that by its oxygen atom (−0.73 eV). This indicates the carbon atom interacts with the Pd atom more efficiently. For NO adsorption, we can see also that the binding of nitrogen atom to the Pd is more stable than that of oxygen atom. For all cases studied, we found that the N_2_O molecule shows a weak chemisorption on the Pd-decorated stanene monolayer compared with the other gas molecules. Besides, the adsorption of all gas molecules in pattern-III, in which the Pd atom was adsorbed on the valley site of stanene, was found to be more energetically favorable than the adsorption in other two patterns. This means the valley site Pd-embedded stanene monolayer possesses superior sensitivity to the gas molecules, and provides more stable configurations. The origin of the chemisorption of gas molecules on Pd-decorated stanene can be reflected by studying their charge density difference plots as shown in Fig. 11. As a matter of convenience, we only presented the CDD plots for CO and NH_3_ adsorbed systems. The charge accumulation on the adsorbed gas molecules indicates the effects of adsorption on the electronic properties of the system. The increase of the electronic density at the middle of the newly formed covalent bonds indicates the chemisorption of CO and NH_3_ molecules on the system.

**Fig. 12:**
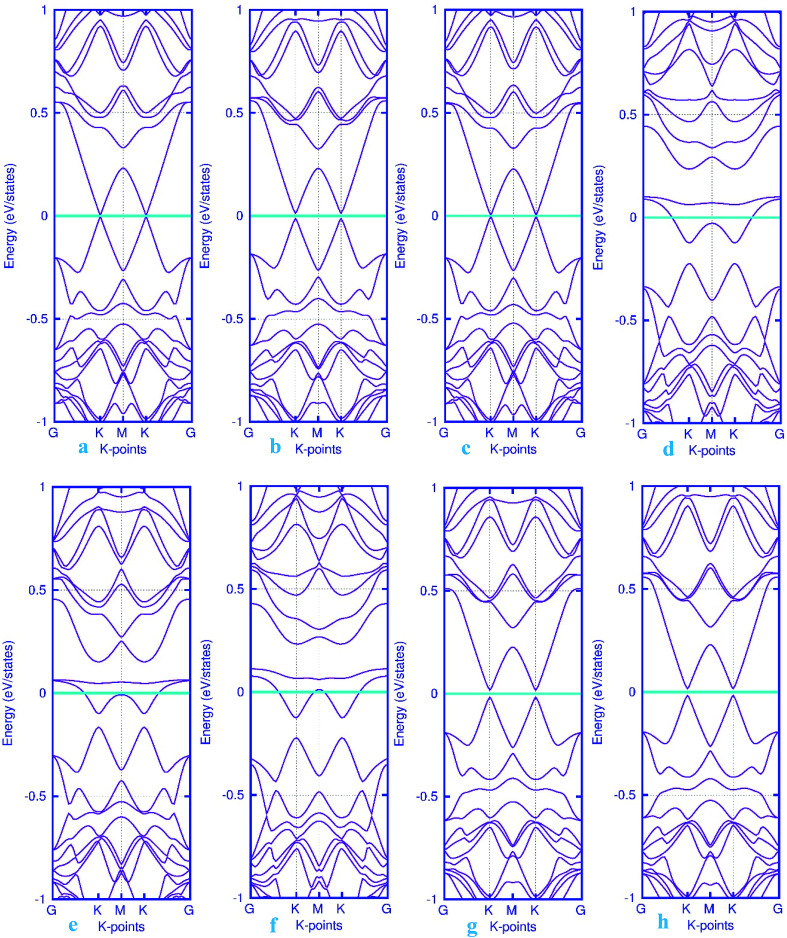
Electronic band structure plots for pattern-III Pd-decorated stanene monolayers with adsorbed CO (a–c) NO (d–f), N_2_O (g) and NH_3_ (h) molecules. The Fermi level is shifted to zero and indicated by a cyan solid line.

Finally, the band structure calculations were performed to further investigate the changes in the electronic structure upon the adsorption of gas molecules. For the non-adsorbed Pd-embedded system, as mentioned above, the system exhibits a semiconductor characteristics with a Fermi level located at K point. The band structure plots for pattern-III Pd-decorated stanene monolayers with adsorbed molecules were displayed in Fig. 12. It can be seen from this figure that except NO adsorbed complex, all systems represent semiconductor characteristics after the adsorption process. Moreover, we examined the electron charge transfer between the gas molecules and substrate using the Mulliken population analysis (see Table 1). The results suggest that the positive charge transfer occurred from the gas molecules to the Pd-decorated stanene monolayers.

## 4. Conclusions

In this paper, we have carried out density functional theory (DFT) calculations to investigate the adsorption of some gas molecules (CO, NO, N_2_O and NH_3_) on the surface of Pd-decorated stanene monolayers. Three different embedding sites for Pd on the stanene surface were considered, and the effects of the adsorption of gas molecules on these Pd-embedded systems were analyzed in detail. The adsorption of Pd atom on the hollow site was found to be more stable than that on the top and valley sites. For CO and NO adsorption on the Pd-decorated stanene, the adsorptions by carbon and nitrogen atoms were more energetically favorable than those by oxygen atoms. Thus, carbon and nitrogen toms of the CO and NO molecules prefer to bind to the Pd atom on the system. The considerable electron orbital overlaps between gas molecules and Pd-embedded stanene confirm this fact that strong chemisorption occurs in these cases. It was found that the positive charge transfer occurred from the gas molecules to the Pd-decorated stanene monolayers. On the basis of our obtained results, Pd-embedded stanene monolayer can hold a great potential as a promising candidate for gas sensing.

## Conflicts of interest

There are no conflicts to declare.

## Supplementary Material
